# A Comprehensive Review on the Efficacy of Several Pharmacologic Agents for the Treatment of COVID-19

**DOI:** 10.3390/life12111758

**Published:** 2022-11-01

**Authors:** Fatma Haddad, Ghadeer Dokmak, Rafik Karaman

**Affiliations:** 1 Pharmaceutical Sciences Department, Faculty of Pharmacy, Al-Quds University, Jerusalem 9103401, Palestine; 2 Faculty of Life Sciences, University of Bradford, Bradford BD7 1DP, UK; 3 Department of Sciences, University of Basilicata, 85100 Potenza, Italy

**Keywords:** COVID-19, antivirals, SARS-CoV-2, immunomodulators, monoclonal antibodies

## Abstract

**Simple Summary:**

Treatments for coronavirus disease-2019 (COVID-19) have utilized a variety of medications, including antivirals, immunomodulators, and other therapeutics such as antibiotics, stem cells, and plasma therapy. Each COVID-19 treatment option has advantages and disadvantages. The therapeutic effects of each medication used to treat COVID-19 patients are discussed in this review. The Food and Drug Administration (FDA) has granted emergency use authorization to some of the proposed treatment medicines for COVID-19 patients. Lopinavir/ ritonavir, favipiravir, ivermectin, nirmatrelvir, interferons, corticosteroids, tocilizumab, sarilumab, siltuximab, canakinumab, colchicine, tofacitinib, thalidomide, convalescent plasma therapy, and mesenchymal stem cells have been shown to have positive effects to treat COVID-19 patients, while anakinra, ruxolitinib, and azithromycin have proven ineffective for COVID-19 therapy; however, no solid conclusion has been obtained from the results of different clinical trials. Therefore, further randomized trials are required to support the validation of their utility. However, most studies concluded that utilizing chloroquine and hydroxychloroquine to treat COVID-19 patients had no effect and advised against doing so. This study helps choose appropriate COVID-19 medications in different nations and advances our knowledge of these drugs’ clinical efficacy.

**Abstract:**

SARS-CoV-2, the coronavirus disease-2019 (COVID-19), and the cause of the pandemic is extremely contagious among people and has spread around the world. Antivirals, immunomodulators, and other medications, such as antibiotics, stem cells, and plasma therapy, have all been utilized in the treatment of COVID-19. To better understand the clinical efficacy of these agents and to aid in the selection of effective COVID-19 therapies in various countries, this study reviewed the effectiveness of the various pharmacologic agents that have been used for COVID-19 therapy globally by summarizing the clinical outcomes that have been obtained from the clinical trials published on each drug related to COVID-19 infection. The Food and Drug Administration (FDA) has authorized the use of remdesivir, paxlovid, molnupiravir, baricitinib, tixagevimab–cilgavimab, and bebtelovimab for the management of COVID-19. On the other hand, most research advises against using chloroquine and hydroxychloroquine to treat COVID-19 patients because they are not beneficial. Although the FDA has given emergency use authorization for some monoclonal antibodies, including bamlanivimab, etesevimab, casirivimab, and imdevimab for managing COVID-19, they are not currently approved for use because the Omicron variant has significantly reduced their in vitro susceptibility. In this study, we also included a wide range of alternative therapy strategies that effectively treat COVID-19 patients, although further randomized studies are necessary to support and assess their applicability.

## 1. Introduction

The coronavirus disease-2019 (COVID-19), a coronavirus, is a brand-new, fatal illness that has just been discovered around the globe [1,2]. In March 2020, the World Health Organization (WHO) declared COVID-19 to be a pandemic illness and a global health emergency [3]. The COVID-19 virus was first reported in December 2019 and immediately spread throughout the world [4]. The coronavirus is a positive single-stranded RNA virus that is a member of the coronavirus beta subgroup and a member of the coronaviridae family [5]. The coronavirus has been compromised by three coronavirus outbreaks: the middle east respiratory syndrome coronavirus, which was discovered in 2012 in Saudi Arabia [6]; the severe acute respiratory syndrome coronavirus (SARS-CoV), which was discovered in 2002 in the Chinese province of Guangdong [1]; and last but not least, the COVID-19 outbreak, which was dubbed the third worldwide outbreak [1,6]. Due to COVID-19’s striking resemblance to SARS-CoV and its symptoms, which include fever, dry cough, breathing problems, headaches, exhaustion, and sore throat with an incubation period of 2–14 days [7], the virus is also known as SARS-CoV-2. In the first 7–10 days after infection, there is a peak in viral replication, followed by the immunological response after 10 days and up to two weeks of the infection [8]. Following the incubation period, COVID-19 individuals experience mild symptoms that last for about 5–8 days [8]. The Coronavirus infects people by attaching its spike glycoprotein to the angiotensin-converting enzyme 2 receptors on the host cells [9].

The viral spike glycoprotein, which is recognized in COVID-19 strains as an antigen, was added to the COVID-19 vaccines that are currently on the market [10]. Pfizer, BioNTech, Moderna, Gamaleya, Novavax, Oxford-AstraZeneca, Sinopharm, Bharat Biotech, Johnson & Johnson, and Sinovac are the companies that make the top nine vaccines [10]. According to recent research, SARS-CoV-2 produces a large missense mutation in the spike protein trimeric, which could increase its transmissibility and compromise the vaccine’s effectiveness [4,11,12]. This development became the main worry over the diminished efficacy of cell-mediated immunity brought on by vaccines due to the emergence of several SARS-CoV-2 mutations [10]. Viruses have the innate potential to alter and develop variants throughout time. While some variants are present and subsequently vanish, others continue [13,14,15]. Numerous COVID-19 variants have been discovered and have rapidly spread over the world, including the South African variants, the Brazilian variant, the United Kingdom variant, the United States Midwest variant, and others [13]. Variants have been proven to alter mortality, enhance transmissibility, delay treatment, and diagnosis, as well as having the capacity to reinfect healthy people and even those who have received vaccinations [13]. The VOC202012/01 strain of B.1.1.7 was discovered in the UK in September 2020 [13]. It was demonstrated to be 40–80% more transmissible than the original strain [13]. Additionally, it was noted that mortality was nearly 55% greater in this variant than in other variants [13,14]. In October 2020, the 501Y.V2 strain of B.1.351 was found in South Africa [13]. This variant was found to be more communicable and, therefore, was most usually seen in young people who did not have underlying illnesses [15]. In December 2020, North Brazil became the site of the first discovery of the P.1 variants, also known as B.1.1.28.1 [13]. Naveca’s et al. investigation indicated that this variant was 2.2 times more contagious and occasionally caused reinfection in individuals who had previously recovered from COVID-19 [13,16]. Due to their similarity in the receptor binding mutations, B.1.351 and P.1 variants were also said to have the same vaccine effectiveness [13]. A novel SARS-CoV-2 variant known as B.1.617.2 (the Delta variant) was first discovered in India between March and May 2021 [13]. The Delta version has been the most predominant SARS-CoV-2 variant seen recently in confirmed COVID-19 cases after spreading to the majority of the world’s countries [17]. Due to its increased transmissibility from the original SARS-CoV-2, it proved to be more pathogenic [18,19]. The “Omicron” variant of SARS-CoV-2, on the other hand, has been classified by the WHO as a new “super variant” and was first discovered in South Africa on 9 November 2021 [20]. Following spontaneous infection and vaccination, omicron has been linked to increased transmissibility and immune evasion [21]. Despite booster doses being administered to increase the neutralizing activity, it has been observed that vaccinated people have much lower levels of neutralizing antibodies to the omicron variant of SARS-CoV-2 than they do to the original strain or the Delta variant [13].

COVID-19 has been treated with a variety of drugs, including antiviral drugs that prevent viral RNA-dependent polymerase (RdRp) or syntheses, viral protein synthesis, or viral entrance [22]. In order to reduce the hyperinflammation that the COVID-19 virus causes, immunomodulators have also been utilized to treat COVID-19 patients [23]. As an illustration, SARS-CoV-2 binds to the alveolar epithelial cells before stimulating the innate and adaptive immune systems and causing the production of a variety of inflammatory cytokines, such as Interleukin-6 (IL-6), TNF-α, and others [23]. Patients experience cytokine storms and severe symptoms as a result, and they eventually develop multiple organ dysfunction [23]. Immunomodulators, such as siltuximab, anakinra, tocilizumab, and corticosteroids, have been suggested and tested as COVID-19 therapies [24]. Some monoclonal antibodies, including bamlanivimab, casirivimab, and imdevimab, have been used in COVID-19 patients to neutralize the virus by preventing the binding of its spike protein and so inhibiting its entrance into human cells [25,26,27,28]. Although there is currently no vaccine or treatment that can completely cure COVID-19, some drugs have shown promise in combating the infection, and some of them have been granted emergency use authorization by the Food and Drug Administration (FDA) because they produce beneficial effects in the treatment of COVID-19 [24,29].

In order to better understand the clinical efficacy of these drugs, this study reviewed the effectiveness of several pharmacologic agents that have been used as COVID-19 therapies around the world. This has been achieved by looking at the clinical trials that were published on each drug regarding COVID-19 infection.

The most recent prospective therapeutic approaches that have been suggested, tested, or approved for use in clinical settings in the therapy of COVID-19 are included below.

## 2. Pharmacologic Agents That Have Been Used as COVID-19 Therapy

### 2.1. Antiviral 

#### 2.1.1. Remdesivir

**Figure 1:**
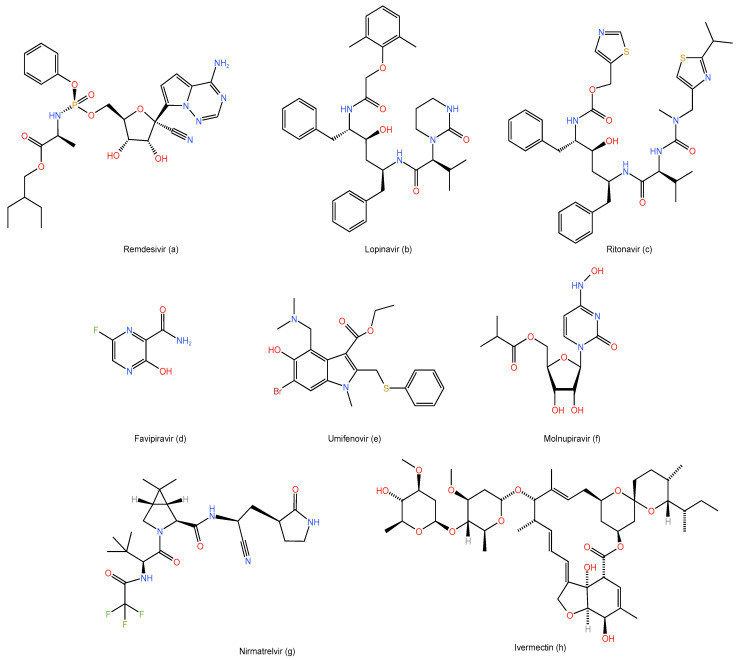
The chemical structures of (**a**) Remdesivir, (**b**) Lopinavir, (**c**) Ritonavir, (**d**) Favipiravir, (**e**) Umifenovir, (**f**) Molnupiravir, (**g**) Nirmatrelvir, and (**h**) Ivermectin are depicted in this diagram.

Remdesivir is an antiviral medication with a broad-spectrum efficacy against RNA viruses (Figure 1a) [8]. It is referred to as an adenosine-C nucleoside prodrug that the host cell metabolizes and converts into nucleoside triphosphate, which blocks viral RNA transcription by inhibiting RdRp [30,31]. It was used to treat viral illnesses such as Ebola [32].

Remdesivir is one of the first antiviral medications that the FDA has licensed (see Table 1). It is administered intravenously to COVID-19 patients [33], and it is advised to take a 200 mg starting dosage followed by a 100 mg dose of remdesivir [8]. This was corroborated by studies conducted in 2020 by Beigel et al. [34], Goldman et al. [35], and Spinner et al., [36] which assessed the usage of remdesivir.

Fifty-three COVID-19 patients have included in a cohort study from three different countries; 68% of those patients improved after receiving remdesivir with oxygen support, whereas 13% of them died [37]. The effectiveness of remdesivir treatment was examined by Wang et al. in a double-blinded, multi-center, placebo-controlled clinical trial on a total of 237 hospitalized patients with severe COVID-19. The study’s overall duration was 21 days, and the patients showed no statistically significant improvement [38]. The National Institute of Allergy and Infectious Diseases conducted a double-blindd, randomized, and placebo-controlled clinical trial on a total of 1062 COVID-19 patients [34]. The study showed that remdesivir had a quick recovery effect after 10 days compared to a 15-day placebo but reported fewer side effects, and the percentage of the death rate fell in contrast to Wang’s study [34,38]. In a randomized open-label multi-center trial, Spinner et al. assessed the effectiveness and side effects of remdesivir, which was given from 5 to 10 days to hospitalized patients with moderate COVID-19 in comparison to the usual therapy [36]. The study, which had 584 hospitalized patients, revealed no changes in the first four days but a substantial difference on day five [36]. In addition to the earlier findings, a meta-analysis of ten clinical trials published in February 2021 showed the effectiveness of remdesivir in improving symptoms and reducing the requirement for oxygen support in COVID-19 individuals who were hospitalized [39]. On the other hand, a WHO-sponsored add-on trial of the global solidarity consortium that was conducted in May 2021 revealed no appreciable impact of remdesivir on the death rate of COVID-19 patients [29]. However, Remdesivir’s impact on the length of hospitalization and death rate was recently examined in Egypt [40]. According to the study, remdesivir usage shortened hospital stays for COVID-19 patients without having any impact on mortality rates [40]. It has been established from multiple trials that remdesivir has antiviral activity in treating COVID-19 patients in contrast with the placebo with reported mild to moderate side effects [29]. If taken along with other drugs, it might be helpful [8]. To establish the role of combination therapy in particular patients, however, more clinical studies are necessary.

#### 2.1.2. Lopinavir/Ritonavir

Lopinavir and ritonavir are oral antiretroviral medications known as protease inhibitors that lessen the amount of virus replication in the host cell (Figure 1b,c) [41]. Human immunodeficiency virus-1 infection in adults, adolescents, and children may be treated with lopinavir/ritonavir when combined with other antiretroviral drugs [42]. Additionally, it can be utilized by medical professionals as a preventative measure against post-exposure illness [43]. This combination’s antiviral efficacy against SARS-CoV was largely identified in the clinical research conducted by Chu et al. [44]. In a 199 COVID-19 hospitalized patient randomized clinical trial, the outcomes of 99 patients receiving lopinavir/ritonavir and 100 patients in the control group were compared [45]. The experiment found no clinical benefit or viral clearance after 14 days of the treatment compared to the placebo group, and only the percentage of the mortality rate was lower in the lopinavir/ritonavir-treated group [45].

Contrarily, an open-label, randomized, phase 2 trial on 86 patients found that adjuvant therapy with lopinavir/ritonavir, interferon beta-1b (IFN-β-1b), and ribavirin reduced viral activity and had a rapid improvement impact on COVID-19 patients [46]. A total of 47 patients with COVID-19 infections were admitted to the hospital in 2020, according to another study that was conducted [47]. In contrast to those in the control group, it was discovered that for patients who had received lopinavir/ritonavir, it had a more pronounced therapeutic effect on lowering body temperature [47]. On the other hand, it was reported in Singapore that treatment with lopinavir/ritonavir did not result in any clinical improvement for hospitalized COVID-19 patients who required oxygen support [48].

As a result, although there were some reported side effects, the combination of lopinavir and ritonavir did not have an impact on the mortality rate. Instead, WHO advised using this combination as adjuvant therapy in conjunction with other drugs to treat COVID-19 [49].

#### 2.1.3. Favipiravir

A novel antiviral medication called favipiravir (Figure 1d) is known as an analog of purine nucleoside that inhibits RdRp and prevents the replication of the RNA virus [50]. The success of favipiravir in combating the Ebola pandemic led to its initial approval in Japan in 2014 [51,52]. It is also effective for treating severe fever with thrombocytopenia syndrome, as well as some antiviral-resistant influenza viruses, such as influenza A [53,54]. A recent in vitro trial against SARS-CoV-2 demonstrated its clinical improvement impact [55]. Additionally, a China-based open-label, non-randomized study found that patients who received favipiravir demonstrated rapid viral clearance, improved chest CT results, and reported fewer adverse effects than those in the other antiviral group. The adverse effects of favipiravir were also reported to be milder than those in the control group [56]. In a different study, 240 COVID-19 patients were involved in total [57]. Favipiravir was given to half of the patients, and Arbidol (umifenovir) was given to the remaining patients (Figure 1e) [57]. The favipiravir group demonstrated faster recovery, although there was no statistically significant difference between the two groups [57]. The effectiveness and safety outcomes of favipiravir for the treatment of COVID-19 patients based on the WHO scale were examined in a meta-analysis of nine studies and a systematic review [58]. Only one of the five studies included in their analysis, which examined the effects of favipiravir for 10–30 days after treatment in five different countries on a total of 827 COVID-19 patients, was deemed non-randomized [58]. According to the meta-findings, these analyses of favipiravir showed a clinical improvement in hospitalized patients over the course of seven days, who had a high level of viral clearance after 14 days and required less oxygen support than the placebo group [58].

Because COVID-19 pneumonia has a significant inflammatory response brought on by a strong, cell-mediated immunity that may inhibit the effectiveness of favipiravir against COVID-19, it is advised to take high dosages of the drug [59]. Another distinguishing trait is the lack of viral production that is favipiravir-resistant [59]. Even though favipiravir did not cause any fatal adverse effects or clinically significant improvement in COVID-19 [60,61], it was confirmed to have favorable effects. To assess the long-term impact of favipiravir, more research is required [62].

#### 2.1.4. Molnupiravir

An oral ribonucleoside analog with a broad antiviral activity that selectively targets RdRp is called molnupiravir (Figure 1f) [63]. Over the years, molnupiravir has proven to be effective against a number of viruses, and it is currently used to treat the COVID-19 virus [64]. Molnupiravir has been given an emergency use authorization by the FDA to be used by the end of 2021 for the treatment of COVID-19 patients who are experiencing symptoms within the first 5 days of the commencement of the infection (Table 1) [65,66]. Molnupiravir, such as remdesivir, affects coronavirus growth and virulence by preventing the virus’s RdRp enzyme from functioning [67,68]. Additionally, the results of the docking study indicate that molnupiravir has an inhibitory effect on the presence of mutations linked to drug resistance since there is a restricted mutation space available in the molnupiravir structure [67]. As a result, it can be utilized and is successful in the treatment of COVID-19 in patients who have developed a resistance to other antiviral medications, such as remdesivir [69]. Molnupiravir should be used twice daily every 12 h between doses of 50 and 1600 mg for a brief length of time (5 days) and in the early stages of infections, according to the pharmacokinetic studies of the drug [69].

Molnupiravir’s impact on SARS-CoV-2 was examined in a number of in vitro studies [67]. For instance, Wahl et al. investigated the inhibitory effect of molnupiravir on SARS-CoV-2 using a study on animal models [70]. Lung-only mice were used in the study, and human lung tissue was grafted to be used eight weeks after surgery in order to evaluate COVID-19 lung infection [70]. Starting between 12 and 48 h after infection, patients were prescribed molnupiravir, which they took every 12 h [70]. The trial found that the lung tissue significantly improved after two days of treatment and that it could be more effective if taken in the early stages of infection [70]. Molnupiravir, which was administered twice daily, was evaluated by Cox et al. for its ability to prevent COVID-19 transmission in ferrets [71]. After 24 h of dosing, the outcomes demonstrated the impact of molnupiravir [71]. An additional examination was conducted in the lung epithelial cells of Syrian hamsters to gauge the effect of molnupiravir on COVID-19 [72]. The outcomes demonstrated that molnupiravir treatment reduced viral replication [72]. In comparison to the placebo group, Abdelnabi et al. assessed the impact of molnupiravir’s dose-dependent effects on the virus titer and RNA load [73]. The molnupiravir medication, which merely delayed the progress of illness without halting viral replication, was tested for its effectiveness [73]. These in vitro experiments reveal that molnupiravir is efficient in treating COVID-19 [73]. On the other hand, the SARS-CoV-2 hamster infection model was used to examine the combination therapy of molnupiravir and favipiravir [73]. The findings showed that molnupiravir and favipiravir combined therapy increased the amount of RNA structural mutations, which decreased the RNA titer [73]. 

There is a risk that molnupiravir, a mutagenic ribonucleoside antiviral medication, will be metabolized by the human host cell and incorporated into the host DNA, leading to mutations [72]. Using the cell model A549-HacCE2, Zhou et al. assessed the impact of molnupiravir and other antiviral medications on SARS-CoV-2 antiviral activity in August 2021 [74]. In animal cells treated with molnupiravir, they reported mutations [74]. A letter was later published in response to the Zhou et al. study stating that there was still no evidence that molnupiravir caused human mutations [75].

The MOVe-OUT research, a randomized, double-blinded, placebo-controlled, phase 3 trial, was carried out to evaluate the impact of molnupiravir on 1433 COVID-19 patients who were not hospitalized and had not received vaccinations [76]. Participants received either 800 mg of molnupiravir or a placebo twice daily for five days [76]. When compared to the placebo group, patients receiving molnupiravir showed a 30% reduction in the mortality of hospitalized patients with early stages of the illness after 3 days of treatment [76]. Oral molnupiravir was successful for the non-vaccinated COVID-19 patients and those who were at high risk of infection development within 5 days of the treatment, according to the trial’s findings, with no clear safety concerns [76]. Additionally, molnupiravir was found to be a successful treatment for COVID-19 infections in a meta-analysis that examined three trials and investigated its impact on a total of 896 COVID-19 patients [77]. Overall, molnupiravir is an effective antiviral for the treatment of COVID-19; however, more in vivo, randomized studies are required to prove its efficacy and safety for the treatment of SARS-CoV-2.

#### 2.1.5. Paxlovid

Nirmatrelvir (Figure 1g) and ritonavir (Figure 1c), a SARS-CoV-2 protease inhibitor that stops coronavirus replication, are combined into paxlovid, an oral antiviral [78]. It was recently developed by Pfizer and received FDA emergency use authorization for the treatment and post-exposure prophylaxis of COVID-19 in December 2021 (Table 1) [78]. The SARS-CoV-2 3-chymotrypsin-like cysteine protease enzyme (M^pro^) is the target of nirmatrelvir [79]. M^pro^ is an antiviral target with a minimal probability of off-target activity that is essential for the viral replication cycle [80]. Nirmatrelvir showed a significant decrease in M^pro^ activity and virus replication across a variety of coronaviruses during in vitro research [79]. Additionally, the in vitro studies demonstrated that CYP3A4 substantially metabolized nirmatrelvir [79].

Nirmatrelvir’s pharmacokinetics were significantly enhanced when co-administered with the CYP3A4 inhibitor ritonavir at a modest dose (100 mg) [81,81]. Nirmatrelvir’s therapeutic benefit will be at its highest since ritonavir slows down the metabolism of the drug, keeping its concentration greater for a longer time [81].

A total of 2246 COVID-19 individuals who were symptomatic, unvaccinated, not hospitalized, and at a high risk of developing severe coronavirus diseases were subjected to a phase 2–3, randomized, double-blinded, and controlled trial [82]. Nirmatrelvir, plus 100 mg of ritonavir, was given to patients at random in a 1:1 ratio, along with a placebo, to be taken twice daily for five days [82]. The viral load, safety, hospitalization due to COVID-19, and death were evaluated through to day 28 [82]. Particularly for those who had a high risk of disease development, paxlovid drastically decreased the likelihood of COVID-19 hospitalization and decreased the mortality rate by 89% when compared to the placebo group [82]. The viral load was reported to be lower with nirmatrelvir plus ritonavir on day 5 of the treatment compared to the placebo, and the incidence of adverse events recorded over the treatment period was comparable in both groups [82].

However, a recent study was conducted on 5287 COVID-19 patients, some of whom had had vaccinations. Using electronic health record data from a significant California healthcare system, the study assessed the impact of paxlovid use on hospital admissions and emergency departments of COVID-19 for 5–15 days and followed a paxlovid treatment course for 5 days. It was reported that paxlovid, when administered as an early-stage medication, lowered hospital admission for mild to moderate COVID-19 individuals who were at risk of the condition developing [83]. As a result, paxlovid is thought to be a viable alternative for treating COVID-19 infection.

#### 2.1.6. Ivermectin

Ivermectin (Figure 1h) is a broad-spectrum antiparasitic medication that has received FDA approval and exhibits in vitro antiviral activity [84,85]. Ivermectin’s clinically beneficial effect on preventing SARS-CoV-2 replication was reported by Caly et al. in an in vitro study [85]. At concentrations greater than the amount utilized in human studies, ivermectin has shown in vitro effectiveness against SARS-CoV-2 [86,87]. Ivermectin was given in daily doses of 300 g/kg for five days to 398 COVID-19 patients in a randomized trial, but no clinical improvement was seen, possibly because using it at low doses could reduce its effectiveness against the infection [88].

Ivermectin was one of the pharmaceutical medicines evaluated in a systemic review and meta-analysis of 110 randomized and observational investigations on COVID-19 patients [89]. Ivermectin was associated with no side effects and a lower death rate in severe cases, according to the results of observational studies that evaluated its effectiveness [89]. In general, the research conclusions demonstrated very little certainty [89].

As an alternative, to assess the treatment effects of ivermectin, a randomized, double-blinded, placebo-controlled trial involving 3515 SARS-COV-2 symptomatic patients was conducted [90]. According to their results, Ivermectin had shown no effects on lowering the frequency of hospitalizations [90]. A recent phase 3, placebo-controlled, double-blinded, and randomized trial [91] also looked into the impact of ivermectin on SARS-COV-2. The trial included 1323 non-hospitalized COVID-19 patients, and 50% of them were vaccinated [91]. Ivermectin had no effect on COVID-19-related hypoxemia, death, emergency visitations, or hospitalization, according to the trial’s data [91]. To further assess the effectiveness and safety of ivermectin on COVID-19 patients, a comprehensive review and meta-analysis of 10 randomized clinical trials were performed on 1173 COVID-19 patients [92]. Ivermectin was shown to have no difference from the placebo group in terms of the mortality rate, length of hospital stays, or reported adverse effects [92]. In addition, a recent meta-analysis and systematic review of 25 randomized clinical trials evaluated the clinical improvement of ivermectin on 6310 COVID-19 patients, with 14 of the trials comparing the drug to a placebo [93]. They discovered that ivermectin had no effect on reducing the probability of mortality or the requirement for mechanical ventilation [93]. Additionally, the evidence for this outcome was not quite clear, despite the fact that there was no increase in the likelihood of severe adverse effects [93].

#### 2.1.7. Interferons (IFNs)

IFNs are cytokines with extensive anti-inflammatory and antiviral activities [94]. The initial line of defense against viral infections is thought to be IFN; however, the downregulation of IFN or a delayed response might lead to additional illness development and viral propagation [95]. In the early stages of COVID-19 infection, when viral load and sickness severity are minimal, IFN type 1 (IFN-I), which includes IFN-alpha (IFN-α) and IFN-beta (IFN-β), is expressed at high levels [96]. Patients with severe COVID-19 infections, on the other hand, seem to have decreased IFN-I expression and higher viral loads in their peripheral blood, which exacerbates inflammatory and pathological responses [96]. However, decreased IFN-I levels in the peripheral blood of COVID-19 patients may act as a sign of the severity of the condition [96]. In response to viral infections, the innate immune system is activated by the cytokines IFN-α and IFN-β [95]. IFN-α is known to diminish inflammatory markers and viral replications, whereas IFN-β is predominantly connected to greater viral clearance [97]. According to two in vitro studies, IFN-α or IFN-β administration is associated with a significant decrease in viral loads and can operate as a preventative measure in the early stages of infection [97]. IFN-β has been shown to be more efficient than IFN-α against SARS-CoV-2, and the majority of studies looked at it as a crucial component of IFN-I-containing regimens [98].

Nebulized interferon-beta-1a (IFN-β-1a) (SNG001) was evaluated on 101 COVID-19 patients in a recent phase 2 randomized clinical trial in comparison to the placebo [99]. SNG001 was associated with quicker recovery and fewer adverse events, according to the WHO Ordinal Scale for Clinical Improvement [99]. The use of IFN-β-1a nebulizers has shown a clinical improvement in hospitalized COVID-19 patients in a recent randomized clinical trial, which was similar to the one described above [100]. Even yet, on day 28 [100], the hospital release rates were comparable between the therapy and placebo groups. In a randomized controlled trial involving patients with severe COVID-19, the effects of IFN-β-1a on death rates were evaluated [101]. After 28 days, the death rate was considerably lower in those who received IFN treatment in addition to hydroxychloroquine, lopinavir/ritonavir, or atazanavir/ritonavir [101]. Another open-label, randomized, phase 2 research revealed that the triple combination group, which received IFN in addition to lopinavir/ritonavir and ribavirin, produced significantly shorter hospital stays than the lopinavir/ritonavir alone group [46].

A randomized, open-label clinical trial showed that IFN-β-1b was safe and effective for treating patients with severe COVID-19 [102]. Patients who received IFN-β-1b had lower incidence rates of frequent and serious side effects than the control group and experienced significantly shorter times for clinical improvement, but there was no statistically significant difference between the hospitalization time and intensive care unit stay between the two groups [102]. IFN-β-1b was demonstrated to have a beneficial therapeutic effect in the early discharge of two Chinese case–cohort studies that assessed the efficacy of different medications for treating COVID-19 [103]. The study also demonstrated that IFN-β-1b and ribavirin co-administration improved clinical outcomes, especially in the first stages of infection [103]. IFN-β-1a and IFN-β-1b were tested against SARS-COV-2 illness in an open-label, randomized controlled experiment [104]. In the early stages of the disease, individuals who received IFN-β-1b in combination with lopinavir/ritonavir or atazanavir/ritonavir with hydroxychloroquine showed a greater improvement [104]. The results indicated that treatment with IFN-β-1a was safer and more effective for treating COVID-19 than treatment with IFN-β-1b [104].

There are still some debates about the therapeutic efficacy of different IFN-α therapies [105], although a phase 2 open-label, randomized clinical trial with moderate COVID-19 patients was used to evaluate the efficiency of pegylated interferon-alpha-2b (IFN-α-2b) [106]. The results confirmed that pegylated IFN-α-2brole accelerated viral clearance and enhanced clinical status on day 15 [106]. Furthermore, a recent case report [107] describing the case of an elderly woman with primary myelofibrosis who consistently tested positive for COVID-19 evaluated the therapeutic improvement of combining ruxolitinib and PEGylated IFN treatment. After four weeks of treatment with pegylated IFNs, the viral RNA was eradicated. This research validates the efficacy of ruxolitinib and IFNs in combined therapy for COVID-19 [107]. An extensive cohort trial that examined the therapeutic efficacy of intramuscular IFN-α-2b (Heberon Alpha R) administration found better rates of recovery and fewer fatalities [108]. According to multi-center cohort analysis, early IFN-α-2b administrations were linked to a decreased mortality rate in COVID-19 patients, but late administration was linked to a greater mortality rate and a delayed recovery [109]. However, the use of IFNs in COVID-19 patients has not been found to be beneficial in other investigations [110,111,112]. For instance, a retrospective cohort trial that examined the effectiveness of an intramuscular injection of IFN-β-1b (betaferon) in hospitalized COVID-19 patients failed to find any benefit [113]. A post hoc analysis was used in a multicenter cohort study on 3808 COVID-19 hospitalized patients to assess the effectiveness of early intramuscular IFN-β administration and its impact on the death rate after 30 days [112]. An early administration of IFN-β had no impact on hospitalized COVID-19 patients, according to the study [112]. As a result, there was no evidence to support the association between early IFN treatment after hospital admission and reduced mortality in COVID-19 patients [112]. Additionally, IFN regimens had no discernible effect on the death rate or length of hospitalization for COVID-19 patients, according to the WHO SOLIDARITY research [111]. A different randomized clinical trial looked into the therapeutic impact of IFNs on COVID-19 hospitalization [110]. In two COVID-19 patient groups with moderate to severe pneumonia, the clinical results were compared [110]. One group received favipiravir plus IFN-β-1b in combination, while the other group received hydroxychloroquine. There were no appreciable differences in the length of hospitalization, admissions to intensive care units, discharges, mortality rates, oxygen saturation at discharge, or changes in inflammatory biomarkers at the time of discharge [110]. In conclusion, a number of studies back up the beneficial effects of IFNs on patients with COVID-19.

### 2.2. Immunomodulators

#### 2.2.1. Corticosteroids

##### Systemic Corticosteroids

Dexamethasone

**Figure 2:**
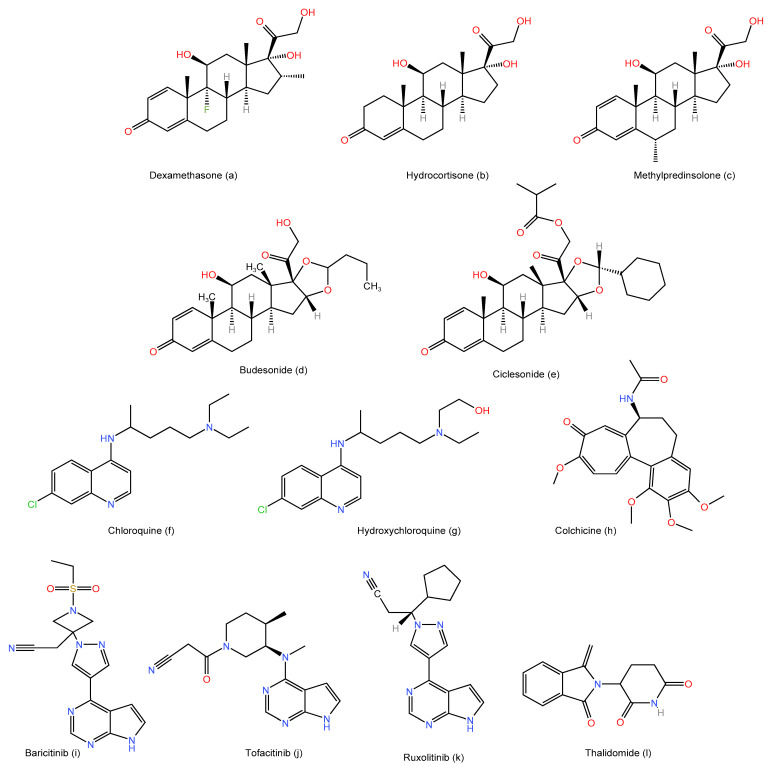
The chemical structures of (**a**) Dexamethasone, (**b**) Hydrocortisone, (**c**) Methylprednisolone, (**d**) Budesonide, (**e**) Ciclesonide, (**f**) Chloroquine, (**g**) Hydroxychloroquine, (**h**) Colchicine, (**i**) Baricitinib, (**j**) Tofacitinib, (**k**) Ruxolitinib, and (**l**) Thalidomide are depicted in this diagram.

Dexamethasone is a corticosteroid with significant anti-inflammatory and immunosuppressive properties (Figure 2a) [114,115]. Numerous inflammatory, autoimmune, and other illnesses have been treated with it [114,115]. Dexamethasone has been studied extensively in clinical trials since the start of the COVID-19 crisis to determine whether it can lower patient mortality [116,117]. Three trials that employed dexamethasone in critically ill COVID-19 patients, as part of a meta-analysis of seven randomized studies that looked into the effects of systemic corticosteroids, came to the conclusion that it was well tolerated and decreased the 28-day death rate in those patients [116]. Dexamethasone was tested in the Randomized Evaluation of COVID-19 Therapy (RECOVERY) experiment to see how it affected hospitalized COVID-19 patients. Of the 6425 participants, 2104 received dexamethasone, while the remaining 4321 were given normal care [117]. Dexamethasone administration for up to ten days decreased the 28-day mortality of patients who needed respiratory support such as mechanical ventilation or oxygen compared to patients who received usual care [117]. However, possible harm could occur in patients who do not need respiratory support [117]. From June 2020 to the end of May 2021, an observational cohort trial was conducted in hospitals run by the United States Department of Veterans Affairs [118]. A total of 19,973 patients were hospitalized in these hospitals within two weeks of receiving a positive COVID-19 test, and within the first two days of their arrival, 15,404 COVID-19 patients were not receiving intensive respiratory assistance [118]. Dexamethasone was given to 34% of these patients who were not receiving oxygen, and it was linked to a 76% increased 3-month mortality rate [118]. Therefore, it was hypothesized that early dexamethasone administration to patients who did not require respiratory support caused injury without improving mortality [118].

Other corticosteroids

Some studies have attempted to assess the efficacy of hydrocortisone (Figure 2b) and methylprednisolone (Figure 2c) on COVID-19 patients, but some of these trials have been terminated early due to enrollments, and no conclusion was reached [119,120,121,122].

##### Inhaled Corticosteroids

Inhaled budesonide

The outcomes of inhaled budesonide (Figure 2d) in COVID-19 outpatients were assessed in two randomized and controlled studies [123,124]. The STOIC trial found that administering inhaled budesonide to adult COVID-19 outpatients reduced the need for urgent medical attention and sped up recovery times [123]. The study had only 146 participants, 73 of whom were given inhaled budesonide and 73 of which received normal treatment. A total of 4700 participants were included in the PRINCIPLE trial, which examined the effects of inhaled budesonide. Of them, 1073 were given the medication, while the remaining participants either received standard medical care alone or received alternative therapies [124]. The PRINCIPLE research discovered the inhaled budesonide decreased recovery times but had no impact on hospitalization or fatality rates [124].

Inhaled ciclesonide

Two randomized studies examined the effects of inhaled ciclesonide (Figure 2e) in outpatients with mild COVID-19 [125,126]. A total of 400 participants were enrolled in the first study, with 197 patients randomly assigned to the ciclesonide group and the remaining participants to the placebo group [125]. They discovered that while utilizing inhaled ciclesonide did not shorten the amount of time it took for patients to report feeling better, these patients experienced fewer additional COVID-19-related hospitalizations or emergency visits [125]. The combination of inhaled and intranasal ciclesonide was explored in smaller research termed CONTAIN, but no appreciable improvement in the symptoms, including fever and/or respiratory symptoms, was seen [126].

#### 2.2.2. Chloroquine and Hydroxychloroquine

The immunomodulatory drugs chloroquine (Figure 2f) and hydroxychloroquine (Figure 2g), which are more potent and less toxic, have been used to treat malaria for many years [127]. Since it has been shown that chloroquine and hydroxychloroquine have in vitro activity against SARS-CoV and SARS-CoV-2, it has been hypothesized that they may be employed as effective therapies for COVID-19 [127,128,129]. As a result, many studies have examined their effectiveness as a potential COVID-19 therapy [130]. However, as neither chloroquine nor hydroxychloroquine has demonstrated clinical efficacy in the majority of trials, it is advised against using them to treat COVID-19 patients [130]. In order to assess the effectiveness and safety of chloroquine and hydroxychloroquine for the therapy of COVID-19 in 61,221 hospitalized patients, a recent meta-analysis examined 42 observational trials and nine randomized controlled studies [130]. They came to the conclusion that neither of the two medications significantly reduced mortality, the time to fever improvement, hospital stay days, the prevalence of mechanical ventilation, or the time for SARS-CoV-2 test conversions that were negative at 1 or 2 weeks [130]. Additionally, both when used as monotherapy and when paired with azithromycin, their use was strongly linked to increased risks of QT prolongations [130,131]. In 1372 non-hospitalized COVID-19 patients, the most recent double-blinded, randomized, controlled research assessed the effectiveness of hydroxychloroquine versus the placebo [132]. According to their findings, the likelihood of hospitalization was not significantly lower in the hydroxychloroquine group compared to the placebo group [132]. The FDA halted its clinical trials on 25 May 2020 because the majority of research warned against the use of chloroquine and hydroxychloroquine in the treatment of COVID-19 patients [22,132].

#### 2.2.3. Colchicine

The care of acute gout, gout prevention, and familial Mediterranean fever are the main conditions for which colchicine (Figure 2h) is utilized as a first-line therapy [133]. Due to its extensive anti-inflammatory activity, it has been demonstrated to possess a prophylactic effect against cardiovascular events in patients with coronary artery disease [134]. Given that it is a well-tolerated medication that is affordable and has anti-inflammatory effects, it has most recently been evaluated as a potential treatment for COVID-19 [135,136]. In 4488 outpatients with COVID-19, a sizable, international, randomized, double-blinded phase 3 research examined the effects of colchicine [136]. There were 2235 patients in the colchicine group and 2253 in the placebo group [136]. Colchicine did not significantly reduce hospitalizations or mortality in those who did not undergo a required diagnostic test [136]. However, patients with positive polymerase chain reaction COVID-19 tests experienced a slight decrease in death and hospital admissions [136]. There were 2755 confirmed COVID-19 outpatients in a different multicenter who underwent randomized, multi-arm, open-label, adaptive platform research [137]. Of all of these patients, 156 were randomly assigned to receive colchicine, 1145 were given standard medical care, and 1454 were given other treatments [137]. When compared to the usual care group, colchicine did not significantly shorten the time before the first self-reported recovery from COVID-19 [137]. A total of 5610 hospitalized COVID-19 patients were randomized to receive colchicine, and 5730 were placed in the usual treatment group in the major, multicenter RECOVERY study [138]. Since no benefit-related 28-day mortality or other secondary outcomes have been found by administering colchicine, the RECOVERY study’s findings do not support the use of colchicine in hospitalized COVID-19 adult patients [138]. The same outcomes were discovered in COLCOVID [139], a different multicenter, which underwent randomized clinical investigation. From a total of 1279 hospitalized COVID-19 pneumonia patients, 640 patients in the COLCOVID trial were given colchicine, while 639 were placed in the usual care group [139]. In the colchicine group, there was no appreciable decrease in mechanical ventilation or 28-day mortality [139]. By contrast, a case–control experiment looked at the impact of colchicine on COVID-19 patients who were hospitalized [140]. In comparison to the control group (n = 78), the colchicine group (n = 34) demonstrated improved results, including decreased mortality, a lower percentage of intubations, and a greater discharge rate [140]. A meta-analysis and systemic review that examined the impact of colchicine using five randomized control trials similarly produced encouraging findings [141]. In their research, they tested 16,048 COVID-19 patients [141]. Of these total patients, 8091 were randomized to receive conventional therapy, while 7957 received colchicine [141]. The colchicine group showed a significant decline in C-reactive protein levels and COVID-19 severity [141]. However, when compared to the group receiving conventional treatment, colchicine had no appreciable impact on D-dimer levels, mechanical ventilation, or death rate [141]. The RECOVERY trial and an additional seven studies with 16,248 patients were examined in another systemic review and meta-analysis [142]. While the RECOVERY trial found no differences in the mortality outcomes between the colchicine and non-colchicine-treated groups, other investigations found that colchicine reduced the mortality risk without having a discernible impact on the likelihood of ICU admissions [142]. More research is necessary to demonstrate the effect of colchicine on clinical outcomes in COVID-19 patients.

#### 2.2.4. Anti-IL-6 Receptor Monoclonal Antibodies

##### Tocilizumab

The FDA has approved the use of tocilizumab, a human monoclonal antibody that inhibits the IL-6 receptor, for the treatment of various autoimmune diseases [143,144]. Numerous meta-analyses of randomized studies have examined the effects of tocilizumab in treating patients with COVID-19 since 2020 and have found that it positively affects the mortality risk [145,146,147,148]. Its impact on mortality, according to certain meta-analyses that included non-randomized studies, is insignificant [149,150]. The effectiveness of tocilizumab in treating COVID-19 patients with severe pneumonia was examined in the randomized, embedded, multifactorial adaptive platform trial for community-acquired pneumonia (REMAP-CAP) [151]. It demonstrated a beneficial impact on extending survival and reducing the need for organ assistance [151]. Tocilizumab significantly decreased mortality without causing secondary infections, according to a meta-analysis study that used 33 trials to examine the impact of tocilizumab and two other immunosuppressants on COVID-19 patients [152]. However, tocilizumab also caused fungal co-infection in those patients. An improvement in survival was seen in patients who received tocilizumab, according to the pivotal COVID-19 trial RECOVERY, which examined the impact of the drug in 4116 hospitalized COVID-19 patients [153]. Despite the fact that all of these trials have shown that tocilizumab significantly reduces mortality in COVID-19 patients, neither the individuals who benefited from its use nor the time frame of the disease’s progression was mentioned [154].

##### Sarilumab

The human IL-6 receptor inhibitor sarilumab has recently been studied as a potential treatment for COVID-19 to lessen the excessive inflammatory immune response [155,156,157,158]. It was first approved for the management of rheumatoid arthritis [155]. Sarilumab’s efficacy was unknown, and some studies and systemic reviews claimed that it would be ineffective in hospitalized COVID-19 patients supplemented with oxygen and that non-significant improvements were obtained [155,156,157]. These studies looked at the effect of sarilumab on a small number of patients with the COVID-19 virus [155,156,157]. On the other hand, 53 patients with severe pneumonia and SARS-CoV-2 were treated with 400 mg of sarilumab intravenously in an observation trial and monitored for at least two weeks. Most medical inpatients experienced a considerable improvement in their clinical results, and the length of their hospital stay was reduced with satisfactory safety [158]. Additionally, the international platform investigators of the REMAP-CAP study looked at the impact of sarilumab on 485 patients with severe COVID-19 pneumonia and compared it to 418 participants in the control group, as well as to 972 patients who were given tocilizumab and 378 patients who were given anakinra [151]. Their findings showed that anakinra was ineffective and that both sarilumab and tocilizumab had the same impact on these patients, improving both survival and shortening the duration of organ support [151]. As a result, additional research is needed to verify the effectiveness of sarilumab in treating COVID-19 patients.

#### 2.2.5. Anti-IL-6 Monoclonal Antibody

##### Siltuximab

Siltuximab, a chimeric monoclonal antibody, binds to IL-6 and stops it from functioning [159]. It has been authorized as a treatment for Castleman’s disease [159]. Siltuximab has been used as a therapy option for COVID-19 patients in several studies, and it has demonstrated a favorable impact on lowering the death rate of such patients [160,161,162]. A few of these trials are currently accepting participants, and their results have not yet been published [161,162,163]. The recommended amount of Siltuximab for these patients is 11 mg/kg as a single dose, with the necessity for a second dose being determined by the patient’s condition as it has a lengthy half-life of around 16.2 days [161]. In COVID-19 patients, it was shown to be a well-tolerated therapeutic choice that improved both survival and respiratory function [164].

#### 2.2.6. IL-1 Receptor Inhibitor

##### Anakinra

Anakinra is a recombinant human IL-1 receptor antagonist which is licensed for the treatment of rheumatoid arthritis and cryopyrin-associated periodic syndrome [165,166,167]. Anakinra was administered as soon as possible to patients admitted to hospitals with moderate or severe COVID-19 pneumonia in phase 3 double-blind randomized investigation known as the SAVE-MORE trial [168]. According to their findings, anakinra patients (n = 405) had a decreased probability of clinical progression to severe respiratory failure and a substantial reduction in 28-day mortality when compared to patients in the placebo group (n = 189) [168]. Anakinra’s impact on hospitalized patients with mild to moderate COVID-19 pneumonia was studied in the CORIMUNO-ANA-1 experiment [169]. A total of 59 patients received anakinra out of a total of 116 participants, while 57 individuals received standard care [169]. Anakinra did not enhance the outcomes, and major side effects occurred in 46% of the patients in the anakinra group as opposed to 38% in the usual treatment group, according to the results of the aforesaid trial, which was discontinued early [169]. As indicated, the REMAP-CAP trial tested three IL-6 inhibitors—sarilumab, tocilizumab, and anakinra—in COVID-19 patients who needed organ support. Anakinra was ineffective, despite the beneficial effects of the other two IL-6 inhibitors, sarilumab, and tocilizumab, on these patients [151]. The CORIMUNO-ANA-1 investigation examined the effects of anakinra on hospitalized patients with mild to moderate COVID-19 pneumonia [169]. Out of 116 participants, 59 patients received anakinra, while 57 patients received standard care [169]. According to the findings of the aforementioned experiment, which was terminated early [169], anakinra did not improve outcomes, and significant adverse effects occurred in 46% of patients in the anakinra group as opposed to 38% in the conventional treatment group. As previously mentioned, the REMAP-CAP trial examined three IL-6 inhibitors in COVID-19 patients who required organ support: sarilumab, tocilizumab, and anakinra. Despite the positive effects of the other two IL-6 inhibitors, sarilumab and tocilizumab, on these individuals, anakinra proved to be ineffective [151]. A recent article reviewed the current clinical evidence on the use of anakinra for the treatment of COVID-19 patients [170]. They reported that in COVID-19 patients that required oxygen but were not on invasive respiratory support, the early receiving of a high dose of anakinra, within about the first 7 days of the symptom’s onset, might be beneficial in enhancing the outcomes [170]. However, no absolute conclusion was obtained due to conflicting results and a lack of sufficient blinded trials [170].

#### 2.2.7. Janus Kinase (JAK) Inhibitors

##### Baricitinib

JAK is suppressed with a little oral medication called baricitinib (Figure 2i). Its use is approved for managing rheumatoid arthritis [171]. It received FDA approval in May 2022 [172] for the treatment of hospitalized patients with COVID-19 infections who require oxygen support, mechanical ventilation, or extracorporeal membrane oxygenation. It is noteworthy that the FDA has approved it as the first immunomodulatory therapy for COVID-19 infection (Table 1) [172]. By preventing SARS-CoV-2 from entering and infecting lung cells, it exerts a direct antiviral impact [173,174]. Baricitinib has been used successfully in numerous randomized and controlled studies in COVID-19 hospitalized patients who require oxygen assistance [175,176,177,178]. The FDA’s approval was backed up by the phase 3, double-blinded, randomized COV-BARRIER research [177]. A total of 1525 hospitalized COVID-19 patients were enrolled in the study that examined the effect of baricitinib; 764 of them received baricitinib, whereas 761 received a placebo [177]. Baricitinib therapy was well-tolerated in those patients and decreased mortality [177]. Following the COV-BARRIER trial design, an exploratory study was conducted to investigate the effects of baricitinib in 101 hospitalized patients who required extracorporeal membrane oxygenation or invasive mechanical ventilation [178]. When compared to the placebo group (n = 50), the baricitinib group (n = 51) showed a substantial decrease in the 28-day mortality rate [178]. A total of 8156 participants in the randomized controlled research (RECOVERY) of 10,852 hospitalized COVID-19 patients in the United Kingdom received usual care plus baricitinib, whereas the remaining participants received only usual care [176]. Although the benefit was less significant than in earlier, smaller studies, baricitinib considerably reduced mortality [176]. In 1033 hospitalized COVID-19 patients, the ACTT-2 randomized, double-blind, placebo-controlled study investigated whether the addition of baricitinib to remdesivir was preferable to remdesivir alone [175]. Particularly in patients who received high-flow oxygen or non-invasive ventilation, the combination treatment group (n = 515) was superior to the remdesivir alone group (n = 518) in shortening recovery times and increasing improvements in clinical status [175]. A total of 1010 hospitalized COVID-19 patients who needed extra oxygen were enrolled in the ACCT-4 study, a global, randomized, placebo-controlled experiment [179]. Baricitinib with remdesivir and a placebo group (n = 516) or dexamethasone, remdesivir, and a placebo group (n = 494) were given to patients at random [179]. By day 29, neither group had required mechanical breathing, but baricitinib was linked with noticeably fewer side effects [179]. Baricitinib is therefore thought considered to be a safe and efficient immunomodulating option for the treatment of COVID-19 hospitalized patients.

##### Tofacitinib

Tofacitinib is an oral, powerful, and selective JAK inhibitor (Figure 2j) [180]. For the treatment of rheumatoid arthritis, psoriatic arthritis, and ulcerative colitis, tofacitinib has been FDA approval [181]. In hospitalized COVID-19 pneumonia patients not receiving mechanical ventilation, the STOP-COVID-19 trial assessed tofacitinib’s effectiveness [182]. A total of 289 patients in total were enrolled from 15 different sites in Brazil; tofacitinib was randomly given to 144 patients, while a placebo was given to 145 others [182]. By day 28, tofacitinib outperformed the placebo in terms of reducing mortality and respiratory failure [182]. In 62 COVID-19 patients, a retrospective trial examined the effectiveness and safety of tofacitinib in the treatment of cytokine release syndrome, a significant side effect of the disease [183]. When compared to the control group (n = 30), the tofacitinib group (n = 32) showed a significantly lower incidence of death, hospitalization to the critical care unit, and lung volume [183]. Additionally, tofacitinib has demonstrated a similar safety profile to the control group [183]. Tofacitinib’s efficacy in the treatment of COVID-19 patients will need to be confirmed by more randomized controlled research.

##### Ruxolitinib

Another oral, small-molecule JAK inhibitor with FDA approval for myelofibrosis, acute graft-versus-host disease, and polycythemia vera is ruxolitinib (Figure 2k) [184,185]. In 41 patients with COVID-19, a modest, prospective, multicenter, randomized controlled phase 2 research examined the impact of ruxolitinib [186]. In comparison to the control group (n = 21), the ruxolitinib group (n = 20) demonstrated numerically faster but not statistically significant clinical improvements [186]. Ruxolitinib had modest toxicities and was safe [186]. The effects of ruxolitinib with standard treatment versus placebo + standard care in hospitalized COVID-19 patients who were not on mechanical breathing or in the intensive care unit were compared in the randomized, double-blind, worldwide, phase 3 study known as RUXCOVID [187]. A total of 287 patients received standard therapy and a modest dose of ruxolitinib (5mg twice daily), as opposed to 145 patients who received a placebo and usual care [187]. Ruxolitinib, however, failed to significantly improve outcomes and had no benefit in the treatment of individuals with COVID-19 [187]. A bigger, randomized study is needed to assess ruxolitinib’s impact on COVID-19 patients.

#### 2.2.8. Thalidomide

A small molecule medication called thalidomide (Figure 2l) has anti-inflammatory and immune-modulating properties [188]. Inflammatory diseases, such as Crohn’s disease, Behcet’s disease, and myeloma, have all been treated with it [189,190,191]. Due to its pleiotropic effects on a variety of biological systems, thalidomide has been suggested for the treatment of COVID-19 patients [192]. A 45-year-old female patient with COVID-19 was treated with 100 mg of thalidomide daily together with twice-daily low-dose methylprednisolone in a case study [193]. The severity of certain COVID-19 symptoms, including lung lesions and exudation, was reduced by thalidomide [193]. The patient’s clinical symptoms improved over the course of three days, and after a week, normal cytokine levels were achieved [193]. Thalidomide’s impact was studied in phase 2, a randomized clinical study on 60 COVID-19 patients who were hospitalized [194]. The other half of the patients were given simply the normal medication, while the other half were given the standard treatment plus 100 mg of thalidomide once a day for two weeks [194]. Thalidomide use decreased the rate of intensive care admission without making a discernible difference in either group’s hospitalization time, need for intubation, or rate of death [194]. To determine the effectiveness and safety of thalidomide for the treatment of COVID-19 infection, further randomized trials are required. While assessing the drug’s safety, thalidomide-induced neuropathy, venous thromboembolism, and other side effects should be taken into account [192].

#### 2.2.9. Canakinumab

A human monoclonal antibody called canakinumab blocks IL-1 beta [195]. Systemic juvenile idiopathic arthritis, Muckle-Wells syndrome, and familial cold auto-inflammatory disease can all be treated with it, according to the FDA [195,196]. Some trials [197,198,199] have proposed and assessed it as a possible cure for COVID-19 infection. Canakinumab’s effectiveness was examined in a double-blinded, randomized, controlled study of hospitalized patients with severe COVID-19 [198]. The study included 39 hospitals from across Europe and the United States [198]. Canakinumab [198] was given to half of the patients (n = 227) in a single intravenous infusion, and the other half (n = 227) received a placebo [198]. On day 29, however, there was no discernible survival improvement in the canakinumab treatment group when compared to the placebo group [198]. Canakinumab’s impact on 45 hospitalized COVID-19 patients was studied in another randomized control experiment [200]. No safety issues were found, and there was no appreciable clinical improvement between the canakinumab group and the placebo group [200]. On the other hand, a study examined its impact on 34 hospitalized patients with mild to severe COVID-19 infection outside of an intensive care unit [197]. Canakinumab was given to a total of 17 patients, while a placebo was given to the same number of patients [197]. Without any significant adverse effects, they documented quick and persistent improvements in oxygenation levels in the canakinumab therapy group [197]. Three of the previously described studies [197,198] were among six trials [199] that examined the impact of canakinumab on a total of 1121 COVID-19 patients [199]. Their meta-analysis found that canakinumab treatment groups showed improved mortality and a decrease in acute inflammation [199]. However, additional randomized studies and meta-analyses are required to establish the value of canakinumab therapy in COVID-19 patients.

#### 2.2.10. Bamlanivimab and Etesevimab

Strong human immunoglobulin G1 monoclonal antibodies against the SARS-CoV-2 surface spike protein that mediates the viral entrance into host cells include bamlanivimab and etesevimab [25,26]. ELI Lilly developed the drug bamlanivimab [26]. Eli Lilly, Junshi Biosciences, and the Chinese Academy of Science worked together to develop etesevimab [26]. The FDA has authorized Bamlanivimab and Etesevimab for use in emergencies to treat COVID-19 in patients who are not being treated in a hospital [26]. This was due to the fact that they had a favorable effect and a high safety profile when used to treat COVID-19 outpatients in the early stages of the pandemic [201,202]. A meta-analysis of eight American trials assessed the effectiveness of bamlanivimab in 13,573 COVID-19 patients, with 9382 patients in the control group and 4191 in the bamlanivimab group receiving monotherapy [203]. Four retrospective cohort studies, two case–control studies, and one randomized control trial made up the studies [203]. They came to the conclusion that the bamlanivimab group had a decreased rate of overall mortality, hospitalization risk, and the development of severe COVID-19 disease [203]. The BLAZE 1 trial’s interim analysis, conducted in 2021, examined the impact of bamlanivimab at three doses (700, 2800, or 7000 mg) and with a placebo in 452 COVID-19 out-patients [201]. The only dose that seemed to hasten viral clearance by day 11 was 2800 mg [201]. Later, 577 COVID-19 outpatients were evaluated as part of the BLAZE 1 phase 2/3 research to compare the effectiveness of bamlanivimab monotherapy, bamlanivimab with estesevimab, and a placebo [202]. Bamlanivimab with estesevimab as a combination therapy effectively lowered the SARS-CoV-2 viral load at day eleven compared to the placebo, whereas bamlanivimab alone did not [202]. In 769 ambulatory COVID-19 patients, a phase 3 component of the BLAZE-1 research evaluated the effectiveness of the FDA emergency use authorization doses of the bamlanivimab and estesevimab combo (700 and 1400 mg, respectively) [204]. The findings showed that the combination therapy with the specified dose decreased COVID-19-related mortality, hospitalization, time to symptom improvement and resolution, and time to viral load reduction [204]. Since the Omicron version greatly reduced the in vitro susceptibility to these monoclonal antibodies when given jointly, they are now not authorized for usage in the United States [205].

#### 2.2.11. Bevacizumab

A human monoclonal antibody with an anti-vascular endothelial growth factor (VEGF) activity is called bevacizumab [206]. Although it has FDA approval for the treatment of systemic cancer [206], it is frequently used off-label to treat retinal conditions such as age-related macular degeneration [207]. Patients with COVID-19 have been found to have significantly high levels of VEGF [208]. Increased tissue hypoxia is caused by elevated VEGF levels in COVID-119 patients, which also cause vascular leakiness, plasma extravasation, and pulmonary edema [209,210]. Additionally, VEGF increases pulmonary inflammation [211]. Bevacizumab has therefore been investigated as a potential therapeutic option for COVID-19 patients [212]. Twenty-six COVID-19 patients were the subjects of a non-randomized, signal-arm clinical trial that was carried out in China and Italy [212]. Patients also received conventional care in addition to a single 500 mg dose of bevacizumab [212]. Between the first day and one week later, a significant improvement in the ratio of partial arterial oxygen pressure to the percentage of inspiration O_2_ (PaO_2_/FiO_2_) was noted [212]. By day 28, the majority of patients were improving their oxygen support status, and no deaths were reported throughout the follow-up period [212]. In two cases, a different study [213] assessed the effectiveness of bevacizumab. Clinical improvements occurred in both patients during the first day, and lung imaging, PaO_2_/FiO_2_, and other measures also improved within a week of admission [213]. None of these two patients experienced any adverse effects or complications from bevacizumab [213]. Randomized clinical trials are required because there are not enough studies or other data to demonstrate bevacizumab’s effectiveness and safety in COVID-19 patients.

#### 2.2.12. Casirivimab and Imdevimab

Two non-competing human immunoglobulin G 1 anti-SARS-CoV-2 monoclonal antibodies, casirivimab, and imdevimab bind specifically to the receptor binding region of the SARS-CoV-2 spike glycoprotein inhibiting viral entry into host cells [27,28]. The FDA has granted emergency use authorization for this mixture for the treatment of COVID-19 [28].

A phase 1–3 clinical trial with 275 COVID-19 outpatients that was double-blinded, randomized, multicenter, and placebo-controlled examined the combination therapy of casirivimab and imdevimab [214].

The clinical effect of the combination with two different doses of 2400 or 1200 mg or placebo was evaluated in a modified randomized phase 3 clinical study by including 2519 patients [215]. In all investigations, casirivimab and imdevimab were well tolerated, reduced the viral load in comparison to the placebo, and lowered hospitalization and mortality in patients with severe COVID-19 cases [214,215]. The intravenous lower dose (1200 mg) in the modified study demonstrated a comparable decrease in the risk of hospital admission or mortality as well as virologic efficacy [215]. Therefore, in the emergency use authorization for this combination, the FDA substituted 1200 mg for the higher dose [215]. In a double-blinded, placebo-controlled clinical trial, the safety and effectiveness of the subcutaneous combination of casirivimab and imdevimab were assessed in 753 COVID-19 patients [214]. The trial contained two assessment phases, the first serving as a preventative measure for close contact with COVID-19 patients who had not yet contracted the disease and the second serving as a therapeutic phase for the affected patient [216]. The findings showed that subcutaneous casirivimab and imdevimab prevented symptomatic COVID-19 and asymptomatic SARS-CoV-2 infection in the previously uninfected person who had been in close touch with a COVID-19 patient. Additionally, it shortened the severity and length of the sickness in the infected patients [216]. On 9785 hospitalized COVID-19 patients, an open-labeled, controlled, and randomized clinical trial [217] evaluated the safety outcome and clinical effect of imdevimab and casirivimab. A total of 4839 patients were randomly assigned to receive casirivimab and imdevimab, while 4946 patients received normal care [217]. Casirivimab and imdevimab significantly decreased the 28-day death rate in seronegative patients when compared to seropositive and seronegative individuals [217]. Mortality, cardiac arrhythmias, thrombosis, and major bleeding were all safety outcomes that did not significantly differ between the groups. Only seven people had extremely negative reactions [217]. The effects of a combination of the subcutaneous antiviral imdevimab and casirivimab on the progression of COVID-19 symptoms from an early asymptomatic SARS-CoV-2 infection were also examined in a randomized, placebo-controlled, and double-blinded phase 3 clinical experiment [218]. The trial involved 314 patients with COVID-19, and the findings showed that treatment with subcutaneous casirivimab and imdevimab antibody combinations vs. the placebo significantly reduced the prevalence of symptomatic COVID-19 over 28 days among asymptomatic SARS-CoV-2 individuals living with infected contact on the same premises [218]. The clinical effectiveness and safety of the repeated monthly doses of subcutaneous imdevimab and casirivimab in a healthy volunteer who was not infected with COVID-19 were recently evaluated in a double-blinded, placebo-controlled phase 1 clinical research [219]. A total of 969 subjects received a placebo or imdevimab and casirivimab at doses of 1200 mg up to six times every four weeks [219]. A monthly dose of 1200 mg of casirivimab and imdevimab administered subcutaneously demonstrated low immunogenicity, was well tolerated, and dramatically decreased the risk of COVID-19 [219]. Although imdevimab and casirivimab should not be used as an alternative to vaccination in immunocompetent individuals, the study’s findings regarding their efficacy and safety profile strongly supported their use as a COVID-19 prophylaxis in those individuals who were not anticipated to have a strong enough immune response to vaccinations [219].

An analysis of a cohort of patients who were eligible to receive monoclonal antibodies looked at whether subcutaneous the casirivimab and imdevimab treatment were statistically and clinically comparable to intravenous casirivimab and imdevimab treatment and whether it was linked to a lower 28-day hospitalization and death rate than nontreatment [220]. The study provided a single dose of 600 mg of casirivimab and 600 mg of imdevimab intravenously or subcutaneously to 1959 mild to moderate COVID-19 patients in order to evaluate the effects of casirivimab and imdevimab [220]. When compared to the placebo, casirivimab and imdevimab were subcutaneously delivered to high-risk outpatients with mild to moderate COVID-19 symptoms, reducing hospitalization and death in results that were comparable to those of intravenous therapy [220]. These results encourage the future growth of subcutaneous monoclonal antibody therapies, particularly in areas with limited staffing and treatment capacity [220]. However, because the Omicron version greatly reduced the in vitro susceptibility of these monoclonal antibodies when given combined, casirivimab and imdevimab are not currently approved for usage in the United States [221].

#### 2.2.13. Ticagevimab and Cilgavimab

The long-acting, completely human monoclonal antibodies ticagevimab and cilgavimab, also known as AZD7442, were found to bind to the SARS-CoV-2 spike protein and kill the virus [222]. Additionally, they affected SARS-CoV-2 variants in vitro [222]. AstraZeneca created this combination [222].

Ticagevimab and cilgavimab’s effectiveness and safety were assessed in two phase-3 trials [222]. In 5197 COVID-19 patients, the Provent phase 3 trial assessed the effectiveness and safety of AZD7442 [222]. In a 2:1 ratio, patients were randomly assigned to receive either 300 mg of AZD7442 intramuscularly (n = 3460) or a placebo (n = 1737) [222]. With no known serious side effects, COVID-19 symptoms were reduced by 82.8% compared to the placebo [222]. Additionally, the Tackle experiment [223], a phase 3 randomized, double-blinded, controlled trial, evaluated the therapeutic benefits and safety of treating severe COVID-19 infection cases in unvaccinated individuals and halting infection progression. Participants (n = 9110) were randomly assigned to receive either a placebo (n = 456) or a dose of 600 mg each of ticagevimab and cilgavimab, which were administered intramuscularly (n = 456) [223]. It was determined that mortality was lower compared to the placebo and that the number of severe COVID-19 cases had dropped by 51% [223]. Additionally, when taken in the early stages of infection, the combination of ticagevimab and cilgavimab demonstrated its long-lasting action in avoiding infection and reinfection [223,224]. The FDA granted AZD7442 emergency approval to be used as pre-exposure prophylaxis against COVID-19 in December 2021 as a result of its favorable and promising effects on COVID-19 (Table 1) [225]. In a recent study, intramuscular clinical effects of AZD7442 were assessed in patients with COVID-19 who were immunocompromised and hospitalized [226]. According to reports, AZD7442 treatment provided protection from both the omicron variant infection and severe COVID-19 infection [226]. Therefore, it is advised to utilize AZD7442 as a preventative measure before exposure in such people [226].

#### 2.2.14. Bebtelovimab

Bebtelovimab (LY-CoV1404) is a recently developed completely human monoclonal SARS-CoV-2 antibody that has shown adequate neutralization effectiveness against all SARS-CoV-2 variations and subvariants, including the Omicron variant [227]. It does this by attaching to the virus’ spike protein. The FDA granted emergency use authorization for bebtelovimab in 2022, allowing it to be used in the early stages of infection, particularly for patients who are at high risk for illness progression (Table 1) [227]. This approval was based on the findings of the phase 2 randomized BLAZE-4 trial [228]. A total of 714 COVID-19 patients in total were randomly assigned 1:1:1 to receive a placebo, bebtelovimab alone, or bebtelovimab plus (bamlanivimab and estesevimab) [228]. Both therapy groups saw considerably shorter times for symptom resolution and lower virus loads [228]. Additionally, bebtelovimab neutralized the omicron isolate (BA.1) in the in vitro experiment [228]. It is important to note that there are still no phase 3 study results available that assess bebtelovimab’s impact on COVID-19 patients.

### 2.3. Others

#### 2.3.1. Azithromycin

**Figure 3:**
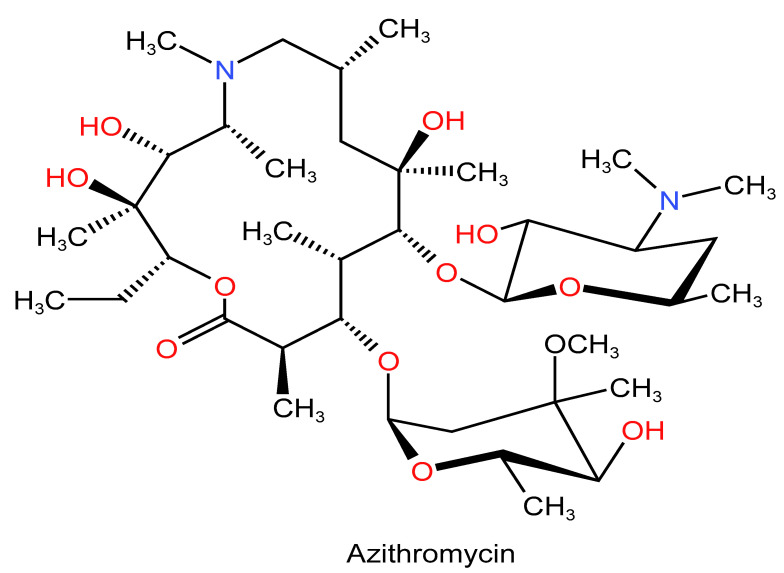
The chemical structure of Azithromycin is depicted in this diagram.

A broad-spectrum antibiotic from the macrolide family, azithromycin (Figure 3), is used to treat a number of bacterial diseases [229,230]. Additionally, it has been shown to have immunomodulatory and antiviral action in bronchial epithelial cells [231,232], which may be useful in viral infections, such as the ongoing global pandemic COVID-19 [233].

Azithromycin was suggested as a possible treatment for COVID-19 infection since it was said to have anti-inflammatory and immunomodulatory effects [234]. As a result, it has been widely utilized to treat the illness in COVID-19’s early stages [231,232]. However, its potential for QT prolongation and cardiotoxicity was the main worry [231,232,235]. Antimicrobial resistance will also develop as a result of its excessive use [236].

The majority of studies [237] were focused on the therapeutic effects of azithromycin in combination with hydroxychloroquine in COVID-19 patients. On 22,984 COVID-19 patients, a meta-analysis and thorough evaluation of randomized controlled trials evaluated the therapeutic benefits and safety of azithromycin [237]. The meta-analysis found that azithromycin had no influence on death rates and that there was no discernible difference in clinical severity, the need for intensive care, hospital admissions, or side effects between individuals treated with or without azithromycin [237]. The systematic review’s conclusions refuted the efficacy of using azithromycin to treat COVID-19 [237]. Furthermore, 263 COVID-19 outpatients were subjected to a single oral dosage of azithromycin in Oldenburg et al. randomized controlled experiments [238]. After 14 days following admission, there was no appreciable difference in self-reported symptoms when compared to the placebo [238]. Azithromycin was found to be ineffective in treating COVID-19 patients [238].

Additionally, the ProPAC-COVID study group conducted a double-blinded, placebo-controlled study to assess the efficacy of azithromycin and hydroxychloroquine in treating hospitalized COVID-19 patients who had positive polymerase chain reaction results [239]. A total of 117 COVID-19 patents were the subjects of the study [239]. The intervention group received a moderate dose of hydroxychloroquine and azithromycin for 15 days, whereas the control group received a placebo and standard therapy [239]. According to their findings, hospitalization and fatality rates were unaffected by the combination of azithromycin and hydroxychloroquine [239].

It is important to note that azithromycin should be used cautiously when treating COVID-19 patients due to its potential pro-arrhythmogenic effects [240]. For patients with COVID-19, additional clinical trials are needed to prove its efficacy and safety.

#### 2.3.2. Convalescent Plasma Therapy (CPT)

Acute infectious illnesses have been successfully treated with CPT over the years [241,242]. There is evidence for CPT therapeutic advantages in COVID-19 according to a number of randomized control clinical trials and meta-analyses [243,244,245,246,247], but other investigations found the opposite [248,249,250,251,252,253]. Donors who have recovered from COVID-19 infections can be used to collect polyclonal CP [254].

A meta-analysis of eight trials assessed the therapeutic impact of CPT on 2341 COVID-19 patients who had been randomly assigned [255]. There was no clinical improvement following CPT in COVID-19 patients, according to the meta-findings analysis [255]. Over sixteen thousand COVID-19 patients were the subjects of a different meta-analysis and systematic evaluation of 16 randomized controlled studies [256]. Their evaluation came to the conclusion that CPT had not improved clinically, and there had been no difference in mortality rates from the placebo [256]. Additionally, 80 patients with severe cases of COVID-19 underwent a phase 2 randomized, controlled, and open-labeled trial to assess the immunological and clinical effects of CP transfusion [257]. In severe COVID-19 patients, CPT did not significantly improve clinical results, and there were no side effects recorded [257].

A randomized, double-blinded, controlled, multicenter research, however, was carried out by Sullivan et al. to assess CPT safety and clinical improvement in COVID-19 patients [258]. A total of 1125 patients were randomly assigned, and 1181 patients had plasma transfusions; the majority of participants were unvaccinated. According to the trial, receiving plasma treatment for nine days reduced the likelihood that a patient would become ill and need to be admitted to the hospital [258].

Two severely ill SARS-CoV-2 hospitalized hematologic cancer patients were the subject of a recent study that analyzed their clinical outcomes [259]. Both patients developed viral spike gene mutations after receiving anti-SARSCoV2 antibody preparations, such as CPT and bamlanivimab [259]. These incidents demonstrate the possibility for SARS-CoV-2 infections to develop antibody resistance in patients with compromised immune systems, making those patients more prone to persistent COVID-19 infection [259].

As there are inconsistent findings about the impact of CPTs on COVID-19 patients, more research is necessary. This should be taken into account for the treatment of the COVID-19 pandemic sickness in the future because antibody levels differ amongst donors [253].

#### 2.3.3. Mesenchymal Stem Cells (MSCs)

Immunomodulatory, anti-inflammatory, and regenerative characteristics are all possessed by MSCs [260]. Comparing different stem cell types, MSCs have the greatest potential for clinical application [261]. In both animal and human clinical investigations, MSC therapy decreased the pathogenic lung abnormalities and suppressed the ineffective immune-mediated inflammatory response brought on by a viral infection [262,263].

Due to its therapeutic effects on acute respiratory distress syndrome [263,264,265,266,267,268], lung fibrosis, and acute lung injury [269], preclinical and clinical research validated the possibility of MSC treatment for COVID-19.

The therapeutic impact and safety of stem cells on patients with COVID-19 were evaluated by a meta-analysis of 17 clinical studies and a systematic review [270]. The trial’s conclusion that stem cell therapy significantly improved clinical outcomes without raising the risk of any documented adverse effects was the primary outcome regarding the safety effects of stem cell treatment [270].

On 100 severe COVID-19 patients with lung damage, a phase 2 double-blind, controlled, and placebo trial evaluated the therapeutic efficacy and safety of human umbilical cord-derived MSCs [271]. After 28 days of treatment, it was stated that the treatment was successful with no known side effects [271]. A phase 2 cohort and prospective trial that evaluated the short- and long-term benefits and safety of umbilical cord-derived MSCs on COVID-19 patients with severe instances were followed with and after a one-year follow-up after umbilical cord-derived MSC treatment [272]. It was determined that umbilical cord-MSC therapy resulted in an improvement in the pulmonary function of COVID-19 patients without any adverse effects being identified [272].

Bone marrow MSC therapy also demonstrated efficacy and safety in severe instances as it indicated an increase in neutrophil and T cell counts and a decrease in the requirement for oxygen support [273]. As a result, MSC therapy appears to be a promising strategy for patients with COVID-19 who have inflammation, lung tissue destruction, and long-term pulmonary impairment [274].

## 3. Summary and Conclusions

There has been a worldwide transmission of the highly contagious SARS-CoV-2 virus. COVID-19 has been treated with antivirals, immunomodulators, and other drugs such as antibiotics, stem cells, and plasma therapy. Each COVID-19 treatment method has benefits and drawbacks. Many COVID-19 varieties, such as the South African version, the Brazilian variant, the United Kingdom variant, the United States Midwest variant, and others, have been found and have rapidly spread over the world. Variants are capable of reinfecting healthy people as well as those who have had immunizations, and they have been shown to modify mortality, increase transmissibility, and delay treatment and diagnosis. This study evaluated the efficiency of numerous pharmacologic substances, such as immunomodulators, antivirals, and others that have been used as COVID-19 therapies around the world, see Table 2. This table can be used as a therapeutic handbook for clinicians and a summary of evidence for pharmacologists.

In conclusion, the FDA has authorized the use of remdesivir, paxlovid, molnupiravir, baricitinib, tixagevimab–cilgavimab, and bebtelovimab for the management of COVID-19. Lopinavir/ritonavir, favipiravir, ivermectin, nirmatrelvir, IFNs, corticosteroids, tocilizumab, sarilumab, siltuximab, canakinumab, colchicine, tofacitinib, thalidomide, CPT, and MSCs have been shown to have positive effects on the treatment of COVID-19 patients, while anakinra, ruxolitinib, and azithromycin are ineffective as COVID-19 therapies; however, no solid conclusion has been obtained from the results of the clinical trials and subsequent randomized studies are needed to warrant the determination of their usefulness.

A few monoclonal antibodies, such as bamlanivimab, etesevimab, casirivimab, and im-devimab, have received emergency use authorization from the FDA for managing COVID-19; however, they are not currently approved for use because the Omicron variant has significantly decreased their in vitro susceptibility. Only tixagevimab–cilgavimab and bebtelovimab have shown neutralizing activities for the Omicron variant and subvariant when compared to other monoclonal antibodies. Additionally, clinical trials for the antimalarial drugs chloroquine and hydroxychloroquine have been suspended due to their lack of effectiveness in treating COVID-19 patients.

## Figures and Tables

**Table 1:** List of drugs that have been granted emergency use authorization by the FDA for the treatment of COVID-19.

Antiviral	Immunomodulator
Remdesivir	Baricitinib
Molnupiravir	Ticagevimab and cilgavimab
Paxlovid	Bebtelovimab

**Table 2:** Summary of the efficacy of several pharmacologic agents for the treatment of COVID-19.

Agent	Classification	Status as COVID-19 Therapy
Remdesivir	Antiviral	Has been granted emergency use authorization by the FDA.
Lopinavir/ritonavir	Antiviral	Positive effects. Further randomized studies are necessary to support the assessment of its applicability.
Favipiravir	Antiviral	Positive effects. Further randomized studies are necessary to support the assessment of its applicability.
Molnupiravir	Antiviral	Has been granted emergency use authorization by the FDA.
Paxlovid	Antiviral	Has been granted emergency use authorization by the FDA.
Ivermectin	Antiviral	Contradictory results. Further randomized studies are necessary to support the assessment of its applicability.
Interferons	Antiviral	Positive effects. Further randomized studies are necessary to support the assessment of its applicability.
Dexamethasone	Immunomodulator	Positive effects. Further randomized studies are necessary to support the assessment of its applicability.
Hydrocortisone	Immunomodulator	Lack of studies. No conclusion was reached.
Methylprednisolone	Immunomodulator	Lack of studies. No conclusion was reached.
Budesonide	Immunomodulator	Further randomized studies are necessary to support the assessment of its applicability.
Ciclesonide	Immunomodulator	Contradictory results. Further randomized studies are necessary to support the assessment of its applicability.
Chloroquine and hydroxychloroquine	Immunomodulators	Clinical trials have been paused due to a lack of benefits for COVID-19 patients.
Colchicine	Immunomodulator	Contradictory results. Further randomized studies are necessary to support the assessment of its applicability.
Tocilizumab	Immunomodulator (Monoclonal antibody)	Positive effects. Further randomized studies are necessary to support the assessment of its applicability.
Sarilumab	Immunomodulator	Positive effects. Further randomized studies are necessary to support the assessment of its applicability.
Anakinra	Immunomodulator	Ineffective. More studies are required.
Baricitinib	Immunomodulator	Has been granted emergency use authorization by the FDA.
Tofacitinib	Immunomodulator	Positive effects. Further randomized studies are necessary to support the assessment of its applicability.
Ruxolitinib	Immunomodulator	Ineffective. More studies are required.
Thalidomide	Immunomodulator	Positive effects. Further randomized studies are necessary to support the assessment of its applicability.
Canakinumab	Immunomodulator (Monoclonal antibody)	Positive effects. Further randomized studies are necessary to support the assessment of its applicability.
Bamlanivimab and etesevimab	Immunomodulator (Monoclonal antibody)	They are not currently approved for use because the Omicron variant has significantly reduced their in vitro susceptibility.
Bevacizumab	Immunomodulator (Monoclonal antibody)	Positive effects. Further randomized studies are necessary to support the assessment of its applicability.
Casirivimab and imdevimab	Immunomodulator (Monoclonal antibody)	They are not currently approved for use because the Omicron variant has significantly reduced their in vitro susceptibility.
Ticagevimab and cilgavimab	Immunomodulators (Monoclonal antibody)	Have been granted emergency use authorization by the FDA.
Bebtelovimab	Immunomodulator (Monoclonal antibody)	Has been granted emergency use authorization by the FDA.
Azithromycin	Other	Ineffective. More studies are required.
Convalescent plasma therapy	Other	Positive effects. Further randomized studies are necessary to support the assessment of its applicability.
Mesenchymal stem cells	Other	Positive effects. Further randomized studies are necessary to support the assessment of its applicability.

## Data Availability

Not applicable.

## Abbreviations

COVID-19	Coronavirus disease-2019
CPT	Convalescent plasma therapy
FDA	Food and Drug Administration
IFNs	Interferons
IFN-I	IFN type 1
IFN-α	Interferon alpha
IFN-α-2b	Interferon-alpha-2b
IFN-β	Interferon beta
IFN-β-1a	Interferon-beta-1a
IL-6	Interleukin-6
JAK	Janus kinase
MSCs	Mesenchymal stem cells
PaO_2_/FiO_2_	Partial arterial oxygen pressure to the fraction of inspiration O_2_
RECOVERY	Randomized Evaluation of COVID-19 Therapy
REMAP-CAP	The Randomized, Embedded, Multifactorial Adaptive Platform Trial for Community-Acquired Pneumonia
RdRp	RNA-dependent polymerase
SARS-CoV	A severe acute respiratory coronavirus infection
SARS-CoV-2	A severe acute respiratory coronavirus infection-2 which is a Coronavirus disease-2019
VEGF	Anti-vascular endothelial growth factor
WHO	World Health Organization
